# Oviposition Deterrence and Larvicidal Activity of Propyl Ether Dillapiole and Piperidyl Dillapiole Against *Aedes (Stegomyia) aegypti* (Diptera: Culicidae)

**DOI:** 10.3390/toxics13040283

**Published:** 2025-04-08

**Authors:** Junielson Soares da Silva, Ana Cristina da Silva Pinto, Samara Silva de Souza, Francisco Célio Maia Chaves, Sabrina da Fonseca Meireles, Rosalina Pinheiro Pereira, Rosemary Aparecida Roque, João Marcelo de Castro e Sousa, Míriam Silva Rafael

**Affiliations:** 1 Postgraduate Program in Genetics, Conservation and Evolutionary Biology, Coordination of Society, Environment and Health, National Institute of Amazonian Research, Manaus 69060-001, AM, Brazil; sabmeireles@gmail.com; 2 Laboratory of Malaria and Dengue, Coordination of Society, Environment and Health, National Institute of Amazonian Research, Manaus 69067-375, AM, Brazil; anacristinadsp@gmail.com (A.C.d.S.P.); rosalinapinheirocavalcante@gmail.com (R.P.P.); rosebio1996@yahoo.com.br (R.A.R.); 3 Laboratory of Ecophysiology and Molecular Evolution, National Institute of Amazonian Research, Manaus 69067-375, AM, Brazil; samsouzabio@gmail.com; 4 Laboratory of Medicinal Plants and Phytochemistry, Embrapa Western Amazon, Manaus 69067-375, AM, Brazil; celio.chaves@embrapa.br; 5 Graduate Program in Pharmaceutical Sciences, Federal University of Piauí, Teresina 64049-550, PI, Brazil; j.marcelo@ufpi.edu.br

**Keywords:** mosquito, insecticidal, *Piper aduncum*, vector control, semi-field, mortality, enzymes

## Abstract

The study of substances of botanical origin is fundamental for the development of new effective alternatives for the control of *Aedes (Stegomyia) aegypti* (Culicidae), a vector of arboviruses in humans. In this study, the potential of two new dillapiole derivatives, propyl ether dillapiole and piperidyl dillapiole, was tested to determine their ability to deter oviposition and their larvicidal and residual effects against *Ae. aegypti* under simulated field conditions, as alternatives for the control of this mosquito. The ability of these substances to deter oviposition by pregnant *Ae. aegypti* females was assessed in the laboratory, and then the larvicide and residual effects of different concentrations were tested under simulated field conditions. The determination of the enzymatic activity in exposed larvae was carried out using sublethal concentrations. The LC_50_ values of propyl ether dillapiole after 24 and 48 h were 24.60 µg/mL and 14.76 µg/mL, and those of piperidyl dillapiole were 31.58 µg/mL and 24.85 µg/mL, respectively. After 48 h of exposure to aged, treated water, the mortality of propyl ether dillapiole (100 µg/mL) and piperidyl dillapiole (200 µg/mL) fell to 81.7% and 75% on the second day, and to 73.3% and 66.7% on the fourth day, respectively. The concentrations of 100 µg/mL of propyl ether dillapiole and 200 µg/mL of piperidyl dillapiole caused oviposition rates of only 3.80% and 4.63% of the eggs of the females, respectively, compared to 22.01% in the negative control (water and DMSO at 2%). In the larvae exposed to propyl ether, piperidyl dillapiole, dillapiole, or the chemical insecticide temephos (positive control), inhibition of acetylcholinesterase (AChE) occurred. Propyl ether dillapiole and piperidyl dillapiole have potential for use as alternative forms of control of *Ae. aegypti*, with propyl ether dillapiole being the most promising molecule. Further studies are needed to understand the effects of these substances on this mosquito and on non-target organisms.

## 1. Introduction

*Aedes* (*Stegomyia*) *aegypti* (Linnaeus, 1762; Diptera: Culicidae) is the main vector of arboviruses DENV, CHIKV, ZIKV, and YFV, which cause dengue, Zika, chikungunya, and urban yellow fever [1]. The use of synthetic chemical insecticides of the organophosphorus class, such as temephos, and pyrethroids, such as deltamethrin, used in vector control campaigns to combat this vector, has caused the selection of certain populations that are resistant to these substances [2].

Plant-based substances have been widely investigated as alternatives for the control of mosquito vectors [3]. Extracts from some plant species have demonstrated ovicidal, larvicidal, pupicidal, and ovisposition deterrent effects against *Ae. aegypti* and other mosquito vectors [4,5,6,7].

Species of the genus *Piper*, such as *Piper aduncum* [8], *P. nigrum* [9], *P. betle* [10], *P. capitarianum* [11], *P. aduncum*, *P. marginatum*, *P. gaudichaudianum*, *P. crassinervium, P. arboreum* [12], *P. cubeba* [13], and *P. tuberculatum* [14], have been investigated for their effects against mosquito vectors of pathogens, including in *Ae. aegypti* [8,10,11,12,13,14,15].

However, there are few studies that have reported the insecticidal effect of substances of botanical origin against *Ae. aegypti* under simulated field conditions. Extracts of *Vitex payos* and *V. schiliebenii* from Kenya were effective against larvae of *Anopheles gambiae* under simulated field conditions [16,17]. Similarly, the formulations of *Annona squamosa* and *A. montana* showed a positive effect against *An. gambiae* and *Culex quinquefasciatus* larvae when tested under the same conditions [18]. *Cryptomeria japonica* essential oil from Taiwan has also been shown to be effective against *An. gambiae* larvae in laboratory and semi-field environments [19]. In Brazil, although species of the genus *Piper* have been extensively tested in the laboratory, only *P. nigrum* had its larvicidal activity against *Ae. aegypti* analyzed in simulated field conditions [20].

*Piper aduncum*, a shrub of the Piperaceae family, has an insecticidal action against *Ae. aegypti* [12,21]. From the essential oil of *P. aduncum*, collected in the region of Manaus, state of Amazonas, Brazil, it is possible to extract dillapiole, which is its major component (50 to 98.9%) [22,23]. In a study by Rafael et al. [8], dillapiole showed a larvicidal effect against *Ae. aegypti*. The effect of this natural molecule was also proven against *An. marajoara* and *Ae. aegypti* [21] and against *Spodoptera frugiperda* [23].

The dillapiole and its semisynthetic derivatives ethyl ether, *n*-butyl ether, methyl ether, propyl ether and isodillapiole had an adulticidal effect on *Ae. aegypti* [24]. Ethyl ether and *n*-butyl ether caused larvicidal and genotoxic effects in *Ae. aegypti* [25] and in *Ae. aelbopictus* [26]. Isodillapiole caused the expression of P450 resistance genes [27] and a genotoxic effect in *Ae. aegypti* [28]. Dillapiole methyl ether also showed ovicidal and larvicidal activity [29] and genotoxic in *Ae. aegypti* [30]. Propyl ether dillapiole and piperidyl dillapiole were toxic to *Ae. aegypti* eggs, larvae and adults [31]. Similarly, the 4-nerolidylcatechol (4-NC) from *P. peltatum* was toxic against *Ae. aegypti*, *Cx. quinquefasciatus*, and *An. darlingi* [32].

Therefore, insecticidal activity (ovicidal, larvicidal, adulticidal, and genotoxic) of dillapiole and some of its semisynthetic derivatives against the mosquito *Ae. aegypti* have been proven in laboratory tests [8,24,25,26,27,28,29,30]. On the other hand, the effects of these substances on this mosquito under simulated field conditions and their mechanisms of action in this insect are unknown.

Some currently used insecticides belong to the class of organophosphates (OPs), whose mechanism of action is based on the inhibition of acetylcholinesterase (AChE) [33]. Additionally, compounds derived from plants with insecticidal potential can affect the AChE of insects [34]. Cholinergic effects, resulting from the accumulation of acetylcholine in the synaptic cleft and subsequent disruption of neuronal signaling, can trigger oxidative stress, leading to genotoxic damage, which can trigger cell death [35].

The exposure of insects to various commercial chemical larvicides and plant-derived substances triggers a series of enzymes that act in metabolic processes fundamental to the normal functioning of the cell [36]. Enzymes such as superoxide dismutase (SOD), catalase (CAT), glutathione peroxidase (GPx), glutathione reductase (GR), and glutathione S-transferase (GST) act in the elimination of reactive oxygen species (ROS) and in the biotransformation and elimination of xenobiotics [37]. Antioxidant enzymes act as a cellular defense mechanism against oxidative stress, eliminating ROS that cause oxidative damage to macromolecules, such as DNA, lipids, and proteins [35,38]. GST plays a key role in the metabolism of xenobiotics and may trigger the development of insecticide resistance [39]. In insects, this enzyme acts in the detoxication of insecticides via the conjugation of glutathione (GSH) in more water-soluble and excretable electrophilic substrates [40].

The activity of these enzymes is little analyzed in mosquitoes, although it can provide important data on the responses of these organisms to the exposure of substances with potential for vector control. Dillapiole derivatives, propyl ether dillapiole, and piperidyl dillapiole, showed ovicidal, larvicidal, and adulticidal effects against *Ae. aegypti* under laboratory conditions [31]. However, to validate the chemical potential as an agent for controlling *Ae. aegypti*, it is crucial to evaluate its efficacy through further tests in conditions closer to the mosquito’s natural environment. Thus, the aim of the present study was to evaluate for the first time the derivatives propyl ether dillapiole and piperidyl dillapiole against *Ae. aegypti* larvae under simulated field conditions.

## 2. Materials and Methods

### 2.1. Acquisition of Dillapiole Derivatives

Propyl ether dillapiole and piperidyl dillapiole were obtained from dillapiole, which was isolated from *P. aduncum* essential oil, according to the method described by Silva et al. [31]. The cultivation of this plant occurred at Embrapa Amazônia Occidental, kilometer 23 on the state highway AM-010 (02°24′52″ S, 54°42′36″ W), in the city of Manaus, state of Amazonas, Brazil.

### 2.2. Maintenance of Aedes aegypti in the Insectarium

Immature *Ae. aegypti* (eggs and larvae) were captured in 2020 in the Coroado district (3°05′38.0”S 59°59 ’02.8” W) in the east of Manaus, Amazonas, Brazil. These were transported to the insectarium of the Laboratory of Cytogenetics, Genomics and Evolution of Mosquitoes (LCGEM), Coordination of Society, Environment and Health (COSAS), Campus I of the National Institute for Amazonian Research (INPA), for identification of specimens and colony formation, according to the method of Silva et al. [31]. The colony was maintained without exposure to any known insecticide, under controlled conditions of temperature (27 ± 2 °C) and humidity (70 ± 5%), with a 12D:12L photoperiod, in which breeding and oviposition occurred [31]. Some of the eggs obtained were used to obtain larvae for bioassays, and the rest were used for the formation of new generations in order to maintain the standard colony.

### 2.3. Acquisition of Aedes aegypti for Bioassays

In the insectarium, six batches of *Ae. aegypti* eggs (n~15,000) (ninth filial generation), with a difference of two days between them, were placed to hatch in a container with drinking water (500 mL). After the hatching of the eggs, the larvae were fed with fish feed (TetraMin^®^ Tropical Flakes, Tetra GmbH, Melle, Germany) until reaching the third instar. These larvae (first batch) were allowed to develop to the adult stage in order to verify the oviposition deterrence effects of the derivatives propyl ether dillapiole and piperidyl dillapiole. The second, third, and fourth batches of larvae were used in the larvicide bioassay in simulated field conditions to determine the lethal acute, chronic, and residual effects of these derivatives. In the fifth batch, the activities of the enzymes catalase (CAT) and glutathione S-transferase (GST) and the total proteins were determined. In the sixth batch, acetylcholinesterase (AChE) activity was measured.

### 2.4. Oviposition Deterrence in the Insectarium

In the LCGEM insectarium, 100 female *Ae. aegypti* (5 to 7 seven days old) from the standard colony, three days after the first blood feeding, were transferred to a cage (30 cm^3^) and fed a 10% sucrose solution. Polypropylene cups (120 mL) lined with filter paper and containing 30 mL of a solution of propyl ether dillapiole (6.25, 25, and 100 µg/mL), piperidyl dillapiole (12.5, 50, and 200 µg/mL), dillapiole at 80 µg/mL as a comparative, temephos (0.012), and two negative controls (NC 1 = water and NC 2 = water + 5% DMSO) were offered for choice of oviposition of the females kept in the cage. The experiment was conducted in duplicate, totaling twenty containers. Every 48 h, the cups were rotated from one location to another within the enclosure. This bioassay was repeated twice using the same conditions. The eggs from each cup were counted under a stereoscopic microscope (Blue edition version Carl Zeiss Stemi 2000, Oberkochen, AxioCam MRc camera, Oberkochen, Germany), and the mean and standard deviation of each concentration and the controls were calculated.

### 2.5. Larvicidal and Residual Effect Bioassay Under Simulated Field Conditions

The larvicidal bioassay under simulated field conditions with propyl ether dillapiole and piperidyl dillapiole was conducted according to guidelines of the World Health Organization (WHO) [41] and Morais et al. [20], with adaptations. The experiment was carried out in April 2023, in the outdoor area of building 31, Coordination of Society, Environment and Health (COSAS), Campus I, INPA, Aleixo, Manaus, Amazonas, Brazil, under local environmental conditions.

At the experimental site, two containers containing 1 L of drinking water each were left to age for 24 h before testing. The batch of third-instar *Ae. aegypti* larvae were acclimatized 12 h before. On the day of the experiment, 3 h before, 20 larvae each were placed in 65 plastic cups (300 mL, totaling 1300 larvae) containing 50 mL of water and about 1 mg of TetraMin^®^ feed. The containers were placed in a steel rack.

The substances propyl ether dillapiole and piperidyl dillapiole (10 mg) were diluted in dimethyl sulfoxide (DMSO) (1 mL). From the stock solution, five concentrations (6.25, 12.5, 25, 50, and 100 µg/mL) of dillapiole propyl ether and five concentrations (12.5, 25, 50, 100, and 200 µg/mL) of piperidyl dillapiole were used. These concentrations were defined in laboratory bioassays based on the LC_50_ values of the substances [31]. Dillapiole used at 80 µg/mL (Rafael et al. [8]) and temephos (0.012 µg/mL) was used as the positive control (PC), as a comparative [42]. The susceptibility of *Ae. aegypti* larvae to these substances was previously confirmed in the laboratory [31]. The negative control (NC) was water and dimethyl sulfoxide (DMSO) at 0,5%. For all the concentrations of substances and controls, five replicates, each consisting of a 300 mL plastic container containing 50 mL of water, were used. Larval mortality was monitored at 24 h and 48 h after exposure to the derivatives, and dead larvae were discarded. After this time, all the larvae were removed from the container.

The analysis of the residual effects on *Ae. aegypti* larvae exposed to aged water treated with propyl ether dillapiole and piperidyl dillapiole began after the larvicidal bioassay (48 h) and lasted 96 h. Five 500 mL containers were prepared for each of the four concentrations of propyl ether dillapiole (12.5, 25, 50, and 100 µg/mL) and piperidyl dillapiole (25, 50, 100, and 200 µg/mL). In addition, dillapiole (80 µg/mL) was used for comparison, along with a positive control (temephos, 0.012 µg/mL), and a negative control. Batches of 20 larvae were added to each container. Two batches of larvae were used (n = 1100). The first (n = 550) was added on the second day (48 h), and the second (n = 550) was added on the fourth day (96 h) after the start of the larvicidal bioassay. The mortality reading for both batches was performed 48 h (2 and 4 days) after the addition of the larvae. The repetition of this procedure was interrupted when mortality reached a minimum of 50%.

The measurement of the ambient temperature (Incoterm 5006—10 °C/+250 °C, Incoterm Thermometer Industry Ltda, São Paulo, SP, Brazil) and pH of the water (Quimis, Q400BC, Quimis Scientific devices Ltda, Diadema, SP, Brazil) was performed every 24 h. The containers remained covered with a nylon mesh to prevent the entry of insects and possible residues during the experimental period.

### 2.6. Sublethal Larvicidal Bioassay of Enzyme Activity

A total of 6000 third instar larvae were used to determine the activity of glutathione S-transferase (GST) and catalase (CAT) enzymes (n = 3000), acetylcholinesterase (AChE), and total proteins (n = 3000). At LCGEM/INPA, larvae, with 100 individuals per replicate (n = 600), were exposed to sublethal concentrations of dillapiole propyl ether (10 µg/mL), piperidyl dillapiole (10 µg/mL), dillapiole (10 µg/mL), temephos (0.001 µg/mL), or the negative control (water and 0.5% DMSO) during 24 h. The larvae were weighed on an analytical balance (Denver Instrumental, APX—153, Sartorius AG, Bohemia, NY, USA) and frozen at −80 °C in a freezer (Panasonic MDF-U56VC-PA, Panasonic Healthcare Company of North America, Wood Dale, IL, USA) until further analysis.

### 2.7. Catalase (CAT), Glutathione S-Transferase (GST), and Acetylcholinesterase (AChE) Activity, and Total Protein Concentration

Analyses of the CAT, GST, and AChE enzymes and the total protein concentration were performed in the Laboratory of Ecophysiology and Molecular Evolution (LEEM) at INPA. For each enzyme assay, analyses were performed using six pools of 100 larvae as biological replicates (n = 6), for each concentration of propyl ether dillapiole, piperidyl dillapiole, dillapiole, temephos, and the negative control. Each replicate consisted of a pool of 100 larvae, and technical replicates (three measurements per sample) were conducted to ensure reproducibility. For analysis of the GST and CAT enzymes, the entire body of the larvae was homogenized (1:4 *w*/*v*) in buffer (20 mM tris-base, 1 mM EDTA, 1 mM dithiothreitol, 500 mM sucrose, and 150 mM KCl, pH 7.6), followed by centrifugation at 9000× *g* for 30 min at 4 °C. For the evaluation of acetylcholinesterase (AChE) activity, the larvae were homogenized (1:3 *w*/*v*) in phosphate buffer (0.1 M, 20% glycerol, pH 7.4) and centrifuged at 12,000× *g* for 20 min at 4 °C. All samples were homogenized in a MultiPro^®^ (Dremel, Racine, WI, USA) tissue homogenizer and centrifuged in a refrigerated centrifuge (Eppendorf, 5430R). The procedure followed protocols for enzyme activity tests [43] and protein purification [44].

The GST and AchE enzymatic activity was determined based on substrate consumption, with no need for a standard curve [42]. Similarly, catalase activity was determined following the method of Beutler [45]. The GST activity was evaluated using 1-chloro-2,4-dinitrobenzene (CDNB) as the substrate [46]. The absorbance was measured using a spectrophotometer (SpectraMax M2, Molecular Devices, San Jose, CA, USA) at 340 nm. The enzyme activity was calculated using the molar extinction coefficient of CDNB (9.6 mM cm^−1^), and the units were expressed as nmol of conjugated CDNB min^−1^ mg^−1^ protein (nmol min^−1^ mg of protein^−1^). The CAT activity was measured via the degradation rate of exogenous hydrogen peroxide (H_2_O_2_), with the generation of oxygen and water, according to the methodology described by Beutler [45]. For the reading, the degradation rate of H_2_O_2_ was measured for 60 s using a spectrophotometer at 240 nm. The results are expressed in µmol min^−1^ mg of protein^−1^.

The AChE activity was determined according to the methodology described by Ellman et al. [47]. After hydrolysis of the acetylthiocholine by the AChE, choline was formed, which was combined with 5,5′-dithio-bis-2-nitrobenzoate (DTNB) to generate a yellow compound measured at 415 nm. The results of the protein^−1^ were expressed as nmol min^−1^ mg^−1^. The total protein content of the body of the larvae was quantified using a spectrophotometer at 595 nm. Bovine serum albumin was used as a standard, as per Bradford [48].

### 2.8. Statistical Analysis

The LC_50_ and LC_90_ values of propyl ether dillapiole and piperidyl dillapiole at intervals of 24 and 48 h were estimated using the Generalized Linear Model (GLM) of concentration–response (Probit) in R software (R Core Team, version 4.4.0, 2024, R Foundation for Statistical Computing, Viena, Austria). The residual effect was analyzed using the Kruskal–Wallis test, followed by Dunn’s test at a 5% probability of error, to detect significant differences between the treatments and controls. The effects of the different compounds on the activity of the GST, CAT, and AChE enzymes were compared by one-way ANOVA, followed by the Tukey test, with a 5% significance level (*p* < 0.05).

## 3. Results

### 3.1. Oviposition Deterrence in Female Aedes aegypti

**Figure 1:**
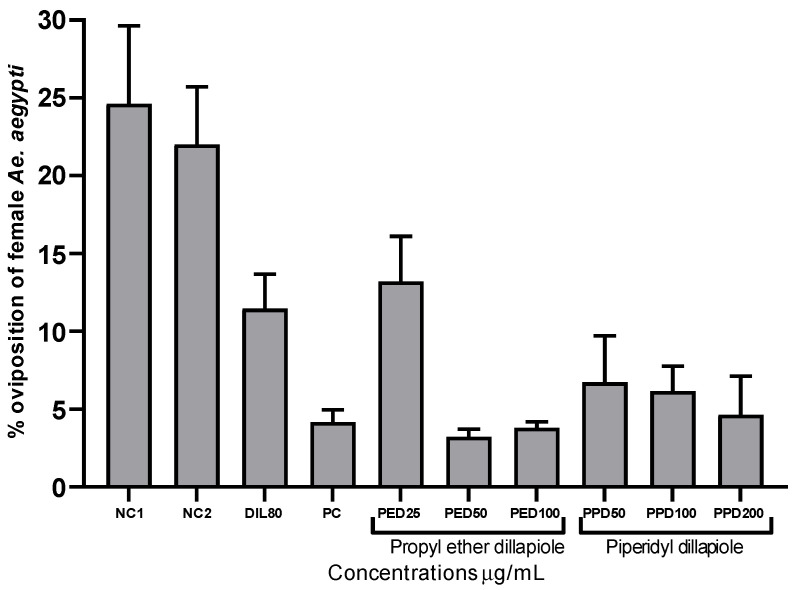
Percentages of oviposition by female *Aedes aegypti* after 7 days in containers, treated with propyl ether dillapiole (PED), piperidyl dillapiole (PPD), negative control 1—NC1 (distilled water), negative control 2—NC2 (water and DMSO at 0.5%), dillapiole (DIL) at 80 µg/mL, and positive control (PC)—temephos at 0.012 µg/mL, evaluated as an oviposition deterrent. The bars represent the mean ± SD. Three replicates (n = 3) were used for each treatment and the control.

In the laboratory experiments, the derivatives propyl ether dillapiole and piperidyl dillapiole, as well as dillapiole at 80 µg/mL and temephos at 0.012 µg/mL, demonstrated significant inhibition (*p* < 0.05) of the oviposition of pregnant *Ae. aegypti* females when compared to the negative controls 1 (water) and 2 (water and 0.5% DMSO), which were 24.62 and 22.01%, respectively. Propyl ether dillapiole, at concentrations of 25 and 100 µg/mL, showed variations of 13.20 to 3.80% in the rate of eggs deposited, respectively. Piperidyl dillapiole, at concentrations of 50 and 200 µg/mL, showed variations of 6.72 to 4.63% in the rate of eggs deposited, respectively (Figure 1). The most significant reduction in oviposition occurred at the highest concentrations of the compounds, notably for propyl ether dillapiole.

### 3.2. Larvicidal Bioassay Under Simulated Field Conditions

**Figure 2:**
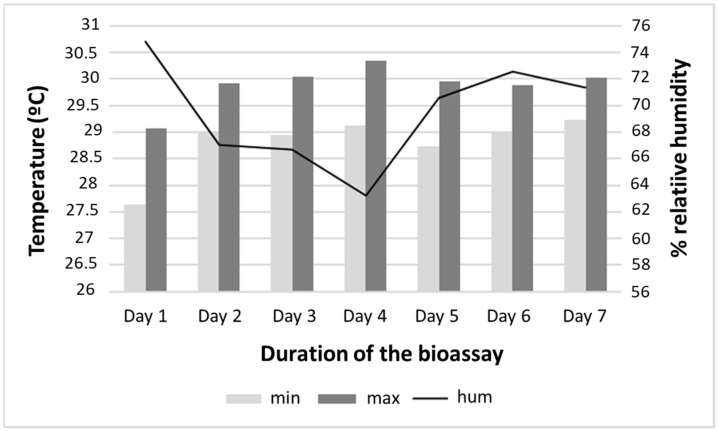
Temperature and humidity of the simulated field conditions at the Instituto Nacional de Pesquisas da Amazônia (INPA), Manaus, Amazonas, during the larvicidal bioassay with *Aedes aegypti*.

Regarding the physical conditions of the environment and the experiment, the average water temperature recorded in the containers throughout the bioassay was 28.7 °C, ranging from 26 to 30 °C. The mean minimum and maximum ambient temperatures at the bioassay site were 28 (24 to 32.6) °C and 29.97 (25 to 33.8) °C, respectively. The pH of the water in the containers ranged from 6.0 to 7.8. The mean humidity was 69.5%, ranging from 63.23 to 74.79% (Figure 2).

**Figure 3:**
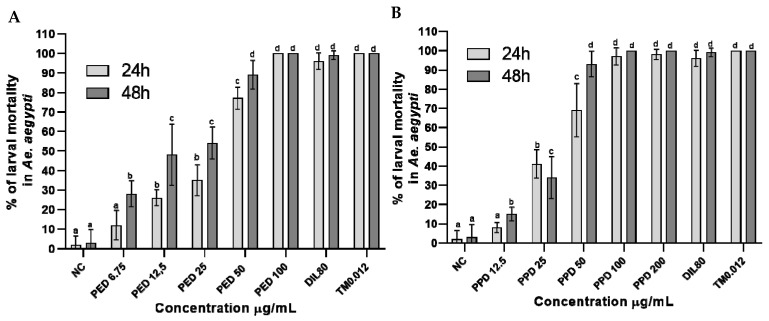
Percentage mortality rates (mean ± SD) of *Aedes aegypti* larvae at different concentrations of dillapiole derivatives, after 24 and 48 h of exposure to propyl ether dillapiole (PED) (**A**) and piperidyl dillapiole (PPD) (**B**). Negative control—NC (water and DMSO 0.5%), dillapiole (DIL) at 80 µg/mL, and positive control—temephos (TM) at 0.012 µg/mL. The bars represent the mean ± SD. A two-way ANOVA was applied, followed by Tukey’s post hoc test with a significance level of 5% (*p* < 0.05), indicated by different letters (a, b, c, d). Three replicates (n = 3) were used for each treatment and the control.

Under the simulated field conditions, the larvicidal activity of the dillapiole derivatives increased with their increasing concentrations. The increased activity of propyl ether dillapiole ranged from 12 to 100% after 24 h and from 28 to 100% after 48 h, at the lowest concentration (6.75 µg/mL) and highest concentration (100 µg/mL), respectively (Figure 3A). Larval mortality in the four highest concentrations of propyl ether dillapiole (200, 100, 50, and 25 µg/mL) was higher (*p* < 0.0001) in relation to the negative control. The mortality of larvae exposed to piperidyl dillapiole ranged from 8 to 100%, at the lowest (12.5 µg/mL) and highest concentrations (200 µg/mL) after 24 h, and from 15 to 100% at concentrations of 12.5 and 100 µg/mL, respectively, after 48 h (Figure 3B).

**Figure 4:**
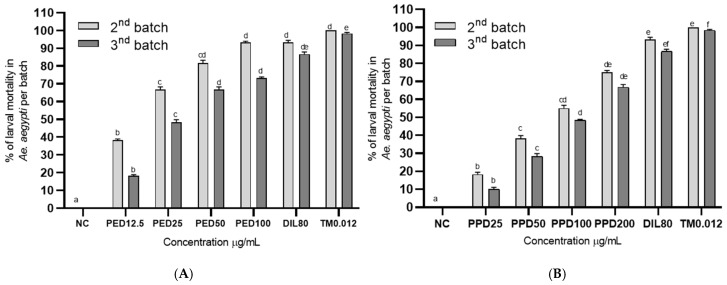
*Aedes aegypti* larvae after 48 h of exposure to aged water treated with propyl ether dillapiole (PED) (**A**) and piperidyl dillapiole (PPD) (**B**). Negative control—NC (water and DMSO 0.5%), dillapiole (DIL) at 80 µg/mL, and positive control—temephos (TM) at 0.012 µg/mL, for 2 (2nd batch) and 4 days (3rd batch). The bars represent the mean ± SD. A two-way ANOVA was applied, followed by Tukey’s post hoc test with a significance level of 5% (*p* < 0.05), indicated by different letters (a, b, c, d, e, f). Three replicates (n = 3) were used for each treatment and the control.

Under the simulated field conditions, propyl ether dillapiole presented LC_50_ values of 24.60 µg/mL and 14.76 µg/mL, after 24 and 48 h, respectively (Table 1). The LC_90_ values were 78.13 µg/mL and 60.85 µg/mL after 24 and 48 h, respectively (Figure 4 and Table 1). For the biolarvicide piperidyl dillapiole, the LC_50_ values were 31.58 µg/mL and 24.85 µg/mL after 24 and 48 h, respectively (Figure 3 and Table 1). The LC_90_ values were 75.22 µg/mL and 49.89 µg/mL for the same exposure times (Table 1).

### 3.3. Residual Effects of Dillapiole Derivatives

The residual activity of propyl ether dillapiole and piperidyl dillapiole under simulated field conditions (Figure 4) was determined by counting and replacing the batch of larvae in the containers every 48 h until significant loss of activity. Both derivatives caused a high mortality rate at the highest concentrations of aged, treated water, after 2 (second batch) and 4 days (third batch), in the residual effect bioassay lasting 96 h. The highest mortality was recorded on the 4th day (second batch of larvae) of exposure to dillapiole propyl ether. On day 2 (second batch), the mortality ranged from 38.3 to 81.7% at the concentrations of 12.5 and 100 µg/mL of propyl ether dillapiole, and from 18.3 to 75.00% at the concentrations of 25 and 200 µg/mL of piperidyl dillapiole, respectively. On day 4 (third batch), the larval mortality decreased to 18.3% and 73.3% at the concentrations of 6.25 and 100 µg/mL of propyl ether dillapiole and to 10 and 66.7% at the concentrations 25 and 200 µg/mL of piperidyl dillapiole, respectively. On days 2 and 4 (second and third batches, respectively), the larval mortality in dillapiole at 80 µg/mL was 93.3 and 86.7 and, in PC (temephos at 0.012 µg/mL), it was 100 and 98.3%, respectively. There was no mortality in the negative control.

Exposure to different concentrations of propyl ether dillapiole significantly affected (χ^2^ = 32.37; df = 5; *p* < 4.998 × 10^−6^) the mortality of *Ae. aegypti* larvae in relation to the negative control. The same pattern was observed for piperidyl dillapiole, for which the mortality rates at concentrations of 50, 100, and 200 µg/mL were statistically (χ^2^ = 33.73; df = 5; *p* < 2.69 × 10^−6^) higher than in the negative control.

### 3.4. Activity of the Enzymes Glutathione S-Transferase (GST), Catalase (CAT), and Acetylcholinesterase (AChE)

**Figure 5:**
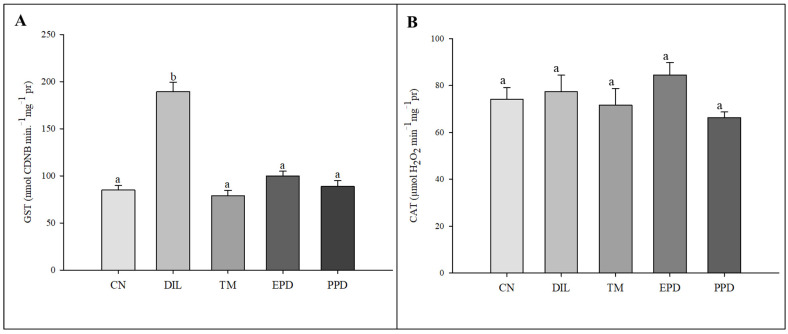
Activity of glutathione S-transferase (GST) (**A**) and catalase (CAT) (**B**) enzymes in *Aedes aegypti* larvae in five experimental groups: NC—negative control; PED—propyl ether dillapiole (10 µg/mL); PPD—piperidyl dillapiole (10 µg/mL); DIL—dillapiole (10 µg/mL); TM—temephos (0.006 µg/mL). Lowercase letters (a, b) indicate significant differences among treatments. Significance level (Tukey test) was *p* < 0.05. Six replicates (n = 6) were used for each treatment and the control.

**Figure 6:**
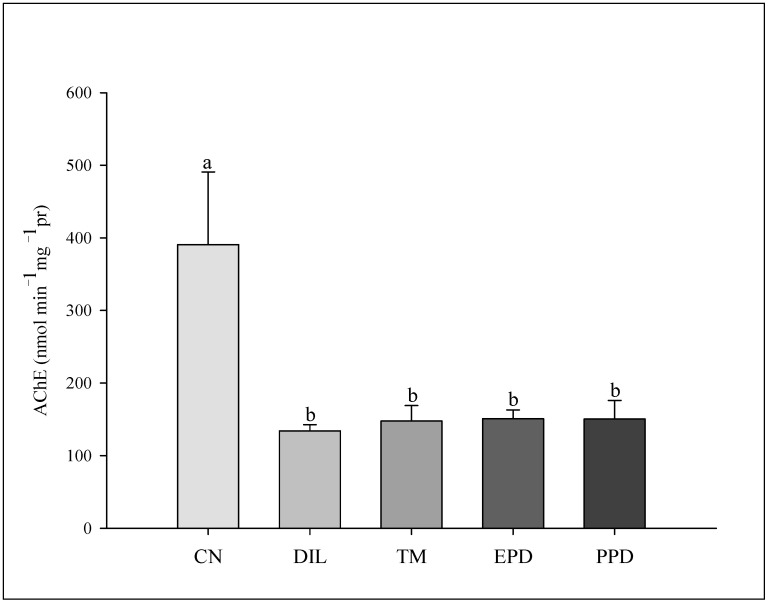
Acetylcholinesterase (AChE) activity in *Aedes aegypti* larvae in five experimental groups: NC—negative control; DIL—dillapiole at 10 µg/mL; TM—temephos at 0.006 µg/mL; PED –propyl ether dillapiole at 10 µg/mL; PPD—piperidyl dillapiole at 10 µg/mL. Lowercase letters (a, b) indicate significant differences among treatments. Significance level (Tukey test) was *p* < 0.05. Six replicates (n = 6) were used for each treatment and the control.

The GST activity increased (F = 47.787; *p* < 0.001) only in the larvae treated with dillapiole at 10 µg/mL. This increase was absent in the larvae exposed to piperidyl dillapiole, propyl ether dillapiole at 10 ug/mL, temephos, and the negative control (water and 0.5% DMSO). The CAT activity remained stable, showing no difference (F = 1.445; *p* = 249) in the treatments in relation to the dillapiole and temephos and the negative control (water and 0.5% DMSO) (Figure 5). Inhibition of AChE activity occurred in all treatments (F = 4.986; *p* = 0.006) when compared to the negative control (Figure 6).

## 4. Discussion

*Aedes aegypti* has been the subject of several laboratory studies involving substances of botanical origin that promote ovicidal, larvicidal, pupicidal, and adulticidal effects, as well as the deterrence of oviposition [6,7,8,11,15,20,25,30]. When studying new larvicides, it is essential to understand the wide range of effects on the target organism in order to determine the frequency of application necessary to achieve effective vector control [49]. However, research that analyzes the different effects of these substances under simulated field conditions is rare.

In mosquitoes, oviposition is a crucial event in the life cycle of these insects [50]. The use of substances that inhibit the oviposition of pregnant females may be an effective alternative to interrupt the life cycle of these individuals and reduce population growth [51]. Studies on the inhibition of oviposition by plant extracts and their derivatives are scarce. The use of substances with insecticidal action in water storage containers deters oviposition in pregnant females, thus reducing the levels of larval populations [5,10,51,52,53].

Essential oils (EOs) with oviposition inhibition activity and larvicidal effect against *Ae. aegypti* are of interest for the control of this mosquito. Laboratory and field tests with piperidines ([1-(3-cyclohexen-1-ylcarbonyl)-2-methylpiperidine] and [1-(3-cyclohexen-1-ylcarbonyl)-piperidine]) demonstrated high oviposition deterrence (43 to 90%) in pregnant *Ae*. *aegypti* and *Ae. albopictus* females [51]. The species *Cuscuta chinensis* strongly inhibited oviposition in *Cx*. *quinquefasciatus* females when compared to the negative control [53]. Similarly, in this study, propyl ether dillapiole and piperidyl dillapiole derivatives inhibited the oviposition of pregnant *Ae. aegypti* females. The deterrent effect of these substances may be related to the capture of chemosensory signals by females of this mosquito [54]. Few studies have reported the insecticidal effect of botanical substances against *Ae. aegypti* under simulated field conditions. However, extracts of *Vitex trifolia* (LC_50_ 76.6 µg/mL) and *V. schiliebenii* (LC_50_ 14.6–17.4 µg/mL) from Kenya were shown to be effective against *An. gambiae* larvae under simulated field conditions [16,17]. Formulations of *Cryptomeria japonica* (LC_50_ 8.22 to 134.84 μg/mL) caused mortality of *An. gambiae* under semi-field conditions [19]. In Brazil, *P. nigrum* and its compound piperine showed larvicidal effects (LC_50_ 0.9–19.03 µg/mL) on *Ae. aegypti* under simulated field conditions [20].

In this study, in simulated field conditions, the larvicidal effect of propyl ether dillapiole and piperidyl dillapiole in *Ae. aegypti* showed that these compounds were toxic, with LC_50_ values of 24.60 and 14.76 µg/mL for propyl ether dillapiole and 31.58 and 24.85 µg/mL for piperidyl dillapiole after 24 h and 48 h, respectively. These derivatives had a larvicidal effect on *Ae. aegypti* in the laboratory, with LC_50_ values of 48.31 and 67.18 µg/mL, respectively after 24 h [31]. Therefore, the derivatives were more toxic when tested under simulated field conditions in comparison with the laboratory tests. Similar results were observed by Silva et al. [46] when testing the larvicidal effects of quinone derivatives, extracted from *Connarus suberosus,* in the laboratory and under simulated field conditions.

Plants of the genus *Piper* present a variety of chemical compounds, including dillapiole, apiol, myristicin, safrole, sarisan, linalool, nerolidol, β-pinene, α-humulene, and β-caryophyllene, among others, which have insecticidal activity against mosquitoes [52,55]. The essential oils of *P. betle* [10], *P. longum* [56], *P. aduncum*, *P. marginatum*, *P. gaudichaudianum*, *P. crassinervium*, and *P. arboreum* have shown larvicidal activity against *Ae*. *aegypti* [12], *P. nigrum* [20] and *P. macedoi* [57] have shown toxic effects against *Ae. aegypti*.

Similarly, *P. nigrum* and its compound piperine caused high mortality in *An. arabiensis*, *An. coluzzii*, *An. gambiae*, *An. quadriannulatus,* and *An. funestus* [9]. The EO of *P. capitarianum*, containing, as its main components, trans-caryophyllene, α-humulene, and β-myrcene, showed larvicidal and adulticidal effects against *Ae. aegypti* and *Ae. albopictus* [11]. The essential oil of *P. purusanum* and its compounds β-caryophyllene, α-humulene, and germacrene D caused ovicidal and larvicidal effects against *Ae. aegypti*, *Ae. albopictus*, *An. albitarsis*, *An. triannulatus*, *An. darlingi,* and *An. nuneztovari*, with inhibition of acetylcholinesterase [15]. Hinokinin, a compound isolated from extracts of *P. cubeba*, showed larvicidal action in *Ae. aegypti* [13]. *Piper tuberculatum* essential oil and its major compound β-caryophyllene (54.8%) demonstrated larvicidal activity (LC_50_ values of 48.61 and 57.20 µg/mL, *p* < 0.05), inhibition of acetylcholinesterase (IC_50_ values of 57.78 and 71.97 µg/mL) and increased production of reactive oxygen and nitrogen species in *Ae. aegypti* larvae [14]. The results of laboratory tests suggest that plant-derived compounds may be an effective alternative for controlling *Ae. aegypti*. However, few of them have been evaluated under simulated field conditions, as was carried out in this study with propyl ether dillapiole and piperidyl dillapiole.

The dillapiole present in the essential oil of *P. aduncum* has shown larvicidal and genotoxic activity [8], as well as adulticide activity [24], against *Ae. aegypti* and toxic effects against *S. frugiperda* [23]. Its derivatives ethyl ether and *n*-butyl ether have also shown ovicidal, larvicidal, and genotoxic effects [25], as well as adulticide effects [24] against this mosquito and against *Ae. albopictus* [26]. Another derivative, isodillapiole, promoted the expression of resistance genes [27] and genotoxic action in *Ae. aegypti* [28]. Methyl ether dillapiole also caused ovicidal and larvicidal effects against this mosquito [29], in addition to genotoxic damage to the genome of this mosquito [30]. The 4-nerolidylcatechol (4-NC) compound from *P. peltatum* was toxic and genotoxic against *Ae. aegypti*, *Cx. quinquefasciatus,* and *Anopheles darlingi* [32].

*Clausena anisata* extract showed larvicidal activity against *Ae. eagypti* in laboratory and in simulated field conditions, with residual effects [58]. Quinone derivatives, isolated from *Connarus suberosus*, showed larvicidal activity and residual effects in laboratory and in simulated field conditions against *Ae. aeypti* [49]. Similarly, under these conditions, the essential oil of *Ocimum kilimandscharicum* caused high larval mortality in *An. gambiae* and *An. arabiensis* [59]. The larvicidal and pupicidal activity of garlic oil has been observed in *Ae. aegypti*, in laboratory and in simulated field conditions [60]. The essential oils of *Cymbopogon nardus* and *Eucalyptus globulus* showed larvicidal activity in *Ae. aegypti* (LC_50_ = 14.46), *An. stephensi* (LC_50_ = 12.85), and *Cx. quinquefasciatus* (LC_50_ = 9.23), in addition to inhibition of oviposition in pregnant females (57.89 to 96.09%) [6].

Tectoquinone showed prolonged larvicidal activity that was higher in field tests (100%) than in laboratory tests (87% to 99%), an effect that was attributed to the environmental conditions [49]. The residual effect is the amount of time that the sample remains active [61]. When isolated from *P. nigrum*, piperine, a molecule containing methylenedioxyphenyl in its structure, demonstrated larvicidal activity against *Ae. aegypti*, both in the laboratory (LC_50_ 19.03 µg/mL, after 24 h) and under simulated field conditions [20]. Asaricin and isoasarone, isolated from *P. sarmentosum,* were highly lethal against *Ae*. *aegypti*, *Ae. albopictus*, and *Cx. quinquefasciatus* and showed strong inhibition of acetylcholinesterase [62].

The absorption of toxic substances by a larva can occur through the cuticle, respiratory siphon or via ingestion and, from there, it can act locally or systemically [63,64,65]. In the body, this causes agitation with random movements, which is followed by lethargy and then death [66].

In physiological terms, mosquitoes have a series of detoxification mechanisms against reactive oxygen species (ROS), which are produced in excess by exposure to toxic agents, such as insecticides and plant-derived substances, to minimize oxidative damage to cellular components [67]. ROS can react with different cellular targets, leading to the formation of oxidative lesions in proteins, DNA, and lipids [68].

Organisms can minimize the impacts of ROS due to the action of primary and secondary enzymatic constituents, as well as non-enzymatic constituents [69]. In insects, GST has been implicated in insecticide resistance [70], sequestering [71], and protection against secondary toxic effects, such as increased lipid peroxidation, induced by exposure to insecticides [72]. Extracts of *Stachytarpheta jamaicensis* increased ROS production in *Cx. quinquefasciatus*, resulting in increased detoxification enzymes and the death of larvae [73].

In the present study, we have presented previously unpublished data on the activity of CAT, GST, and AChE enzymes in *Ae. aegypti* larvae after 24 h of exposure to propyl ether dillapiole, piperidyl dillapiole, and dillapiole. There was an increase in GST activity only in subjects treated with dillapiole, with no change in the catalase activity in any of the treatments (Figure 6). The increase in GST activity in *Ae. aegypti* larvae treated with dillapiole suggests a possible enzyme response during the metabolism of this compound. The levels of this enzyme increased in *Amblyomma sculptum* (Acari: Ixodidae) [74] and *Ae. aegypti* [75,76] exposed to dillapiole. There are no records in the literature on the levels of detoxifying enzymes (CAT, GST, and AChE) in *Ae. aegypti* treated with dillapiole derivatives. Therefore, this is the first study to analyze the effects of propyl ether dillapiole and piperidyl dillapiole derivatives in this mosquito. The absence of an increase in enzymes in larvae treated with propyl ether dillapiole and piperidyl dillapiole suggests that they may not induce oxidative stress or be metabolized by other pathways.

A previous study showed that the differential expression of GST family genes is related to the resistance of *Ae. aegypti* to different classes of insecticides, such as DDT, OPs, and pyrethroids [77]. GST enzymes play an important role in phase II detoxification mechanisms in insects [78]. According to Edwin et al. [79], the increase in GST activity in *Ae. aegypti* exposed to plant extracts with insecticidal potential indicates the activation of the detoxification process. Lima et al. [27] demonstrated an increase in the expression of the GSTE7 gene in the first generations of *Ae. aegypti* exposed to the lowest concentrations of isodillapiole. Johnson et al. [34], suggested that the increase in GST activity in flies exposed to lemongrass (*Cymbopogon citratus*) extracts is a response to the active components of the plant.

The presence of methylenedioxy in the dillapiole molecule can inhibit the activity of cytochrome P450 of the insect, thus affecting the phase I metabolism of xenobiotics [80,81,82]. In this research, the activity of enzymes in the P450 complex was not evaluated; however, as propyl ether dillapiole and piperidyl dillapiole also have methylenedioxy in the structure, inhibition would be expected.

CAT did not show a response that was similar to what was observed for GST (Figure 6). Changes in the activity of this enzyme occurred in *Cx. quinquefasciatus* exposed to extracts of *Hyptis suaveolens* [83]. The exposure of *Callosobruchus chinensis* and *C. maculatus* to *Boswellia carterii* essential oil altered CAT activity according to the concentration [84]. Johnson et al. [34] also reported contrasting results between GST and CAT in *Drosophila melanogaster* exposed to *C. citratus* extracts, with an increase in GST activity and a decrease in CAT activity being observed. Catalase is an enzyme that degrades hydrogen peroxide (H_2_O_2_) into water (H_2_O) and oxygen (O_2_) [85] after exposure of the organism to stressful situations, such as exposure to insecticides [86]. The absence of effects on CAT activity observed in *Ae. aegypti* larvae exposed for 24 h to the substances tested may have been due to the exposure period or indicative of an absence of excess H_2_O_2_. Our hypothesis is that under these conditions, the *Ae. aegypti* developed a protective strategy to prevent oxidative stress after exposure, which may explain the non-activation of catalase for hydrogen peroxide degradation.

However, in the present study, AChE inhibition occurred in *Ae. aegypti* larvae exposed for 24 h via both the organophosphorus temephos, and by dillapiole and its derivatives propyl ether dillapiole and piperidyl dillapiole. AChE is an enzyme whose function is to catalyze the hydrolysis of the neurotransmitter acetylcholine [87]. The inhibition of this enzyme by organophosphorus insecticides, including temephos and plant-derived substances, causes the accumulation of acetylcholine, leading to neurotoxic disorders, such as involuntary muscle contraction and paralysis, which can cause the death of the insect [88,89].

The mechanism of action of the derivatives propyl ether dillapiole and piperidyl dillapiole is completely unknown. This is the first study to analyze the effect of these substances on the nervous system of *Ae. aegypti*. The inhibition of AChE induced by these substances suggests an effect on the cholinergic system of this mosquito; however, further analysis is needed to elucidate the mechanism of action of the compounds. The inhibition of AChE in *Ae. aegytpi* after exposure to extracts derived from plants of the genus *Piper* has already been reported [15]. The EO of *P. alatipetiolatum* (Piperaceae) showed larvicidal activity (LC_50_ of 4.53 μg/mL) in *Cx. quinquefasciatus*, with changes in the levels of detoxifying enzymes [90]. *P. tuberculatum* (Piperaceae) EO and its compound β-caryophyllene demonstrated larvicidal activity (LC_50_ values of 48.61 and 57.20 μg/mL), with increased production of reactive oxygen species [14]. Therefore, this effect observed in the larvae of *Ae. aegypti* exposed to dillapiole and its derivatives indicates the neurotoxic potential of these substances, which should be explored.

## 5. Conclusions

The novel derivatives propyl ether dillapiole and piperidyl dillapiole inhibited the oviposition of pregnant *Aedes aegypti* females, which, under simulated field conditions, showed concentration-dependent larvicidal action and little prolonged residual effects, in addition to the inhibition of AChE activity. These effects were more evident in individuals treated with propyl ether dillapiole, demonstrating it to be the most promising molecule for the effective alternative control of this mosquito. However, further studies are needed to elucidate different effects of these substances on the mosquito and on non-target organisms.

## Figures and Tables

**Table 1:** Lethal concentrations (LC_50_ and LC_90_) and 95% confidence intervals of propyl ether dillapiole and piperidyl dillapiole biolarvicides against Aedes aegypti, under simulated field conditions, after 24 and 48 h.

Reading Intervals	LC_50_ (CI 95%) µg/mL	LC_90_ (CI 95%) µg/mL	χ^2^ (df)	Slope ± SE
**Propyl ether dillapiole**				
24 h	24.60 (20.17–30.02)	78.13 (58.87–118.91)	19.81 (3)	2.55 (0.48)
48 h	14.76 (11.21–18.58)	60.85 (44.18–100.76)	15.38 (3)	2.08 (0.40)
**Piperidyl dillapiole**				
24 h	31.58 (26.50–37.34)	75.22 (60.47–103.55)	3.73 (3)	3.40 (0.28)
48 h	24.85 (21.43–28.69)	49.89 (39.11–61.94)	21.87 (3)	4.64 (1.07)

## Data Availability

The data resulting from the findings are available in this study. If necessary, additional information can be provided upon request from the corresponding authors.
